# Stress Controllability Modulates Basal Activity of Dopamine Neurons in the Substantia Nigra Compacta

**DOI:** 10.1523/ENEURO.0044-21.2021

**Published:** 2021-06-15

**Authors:** Li Yao, Yongfeng Li, Zhijun Diao, Yuanyuan Di, Meilin Wu, Chunling Wei, Zhaoqiang Qian, Zhiqiang Liu, Jing Han, Juan Fan, Yingfang Tian, Qiaohua Zheng, Wei Ren

**Affiliations:** 1 MOE Key Laboratory of Modern Teaching Technology, Shaanxi Normal University, Xi’an 710062, China; 2 Faculty of Table Tennis, Badminton and Tennis, Chengdu Sport University, Chengdu 610041, China; 3 College of Acupuncture and Massage, Shaanxi University of Chinese Medicine, Xianyang 712046, China; 4 College of Life Sciences, Shaanxi Normal University, Xi’an 710062, China; 5 School of Psychology, Shaanxi Normal University, Xi’an 710062, China

**Keywords:** basal activity, controllability, dopamine, Stress, substantia nigra compacta

## Abstract

Prolonged stress induces neural maladaptations in the mesolimbic dopamine (DA) system and produces emotional and behavioral disorders. However, the effects of stress on activity of DA neurons are diverse and complex that hinge on the type, duration, intensity, and controllability of stressors. Here, controlling the duration, intensity, and type of the stressors to be identical, we observed the effects of stressor controllability on the activity of substantia nigra pars compacta (SNc) DA neurons in mice. We found that both lack and loss of control (LOC) over shock enhance the basal activity and intrinsic excitability of SNc DA neurons via modulation of I_h_ current, but not via corticosterone serum level. Moreover, LOC over shock produces more significant enhancement in the basal activity of SNc DA neurons than that produced by shock per se, and therefore attenuates the response to natural reward. This attenuation can be reversed by control over shock. These results indicate that although chronic stress per se tends to enhance the basal activity of SNc DA neurons, LOC over the stressor is able to induce a larger enhancement in the basal activity of SNc DA neurons and produce more severe behavioral deficits. However, control over stress ameliorates the deleterious effects of stress, highlighting the role of stress controllability.

## Significance Statement

The impact of stress on the DA system significantly modifies immediate and guides future behaviors. Stress does not have unitary effects on VTA DA neurons, but the effects of stress controllability on substantia nigra pars compacta (SNc) DA neurons are unclear. The present work studied the effects of controllability on the activity of SNc DA neurons by controlling the duration, intensity, and pattern of footshocks to be identical. The results show that loss of control (LOC) over shock produces a larger enhancement in the basal activity of SNc DA neurons and worse behavioral deficits than what caused by uncontrollable shock per se. The results demonstrate the critical role of stress controllability in modulating activity of SNc DA neurons and inducing behavioral deficits.

## Introduction

Severe or prolonged stress will induce maladaptations in a series of critical brain circuits and produce cognitive, affective, and motivational deficits related to neuropsychiatric disorders, such as anxiety and depression, in which the role of midbrain dopamine (DA) system has been the focus of attention (Tye et al., 2013; Hollon et al., 2015). DA neurons of substantia nigra pars compacta (SNc) and ventral tegmental area (VTA) in the midbrain DA system play key roles in stress-related changes of emotional and motivational behaviors (Der-Avakian and Markou, 2012; Schultz, 2013; Tye et al., 2013), and their activities have been associated with a variety of brain functions in response to the signal pertaining to reward and stressor (Ungless et al., 2010; Lammel et al., 2014).

It has been demonstrated that DA neurons in both SNc and VTA respond to natural rewards fairly homogeneously by increasing their firing activity (Cohen et al., 2012; Marinelli and McCutcheon, 2014). Conversely, responses of DA neurons to stressors are more heterogeneous. In monkey, SNc DA neurons are excited by stressors, but those in VTA are more likely to be inhibited (Matsumoto and Hikosaka, 2009; Marinelli and McCutcheon, 2014). The results concerning reaction of VTA DA neurons to stressors still remain controversial. One line of previous studies have found that chronic unpredictable mild stress strongly decreases DA neuronal firing activities in VTA and depresses DA release in the nucleus accumbens (NAc; Moore et al., 2001; Cabib and Puglisi-Allegra, 2012). In contrast, another line of studies have discovered that both chronic restraint and social defeat stress increase DA neuron firing in VTA (Cao et al., 2010; Valenti et al., 2012). In those situations, the firing activity of DA neurons may hinge on the type, duration, intensity, and controllability of stressors and manifest diverse and complex reactions (Joëls and Baram, 2009; Hollon et al., 2015).

In order to evaluate the influence of controllability on the firing activity of midbrain DA neurons, the present study employed the loss of control (LOC) model, in which the duration, intensity, and type of the stressor were identical, but the controllability of the stressor was very different, in different groups of mice. Mice in the LOC group firstly acquired and then lost control over footshocks. Each mouse in the LOC group was yoked with a mouse of the L-Yoked group receiving the identical footshocks. The third group was a positive control group receiving escapable shocks (ES) and acquiring control over shocks in the whole process of the experiment (Yao et al., 2019). The fourth group was a control group receiving no shocks (NS). By using this experiment design, the effects of controllability over shocks can be separated from other parameters of the shocks. Our previous work has shown that, the LOC group exhibited more significant depression-like behaviors than the L-Yoked group, whereas the ES group showed no obvious depression-like behaviors, as compared with the NS group (Yao et al., 2019). Since this LOC model is based on instrumental learning in which SNc is strongly implicated, in the present study the firing activity of SNc DA neurons was observed. We expected that after the stress procedures the firing activity of DA neurons might be reduced in the L-Yoked group but increased in the LOC group. The present work aims to test for this expectation.

## Materials and Methods

### Subjects

Male C57BL/6J mice (from the Model Animal Research Center of Nanjing University, China) and DAT-Cre mice (B6.SJL-Slc6a3^tm1.1(cre)Bkmn^/J; stock #006660) weighing 20–30 g were housed in standard Specific Pathogen Free facilities on a 12/12 h light/dark cycle (lights on at 8 A.M., lights off at 8 P.M.). Food and water were available *ad libitum*, except for the period when mice underwent sucrose preference test (SPT). All animals were maintained in a temperature (23 ± 1°C) and humidity (50 ± 10%) controlled room and gently handled (5 min each day for at least 5 d) before experiment, to minimize manipulation-related stress.

All experimental procedures were conducted during the light phase of the cycle and approved by the Animal Care and Use Committee in accordance with Governmental Regulations of Laboratory Animals of China.

### LOC model

**Figure 1.**
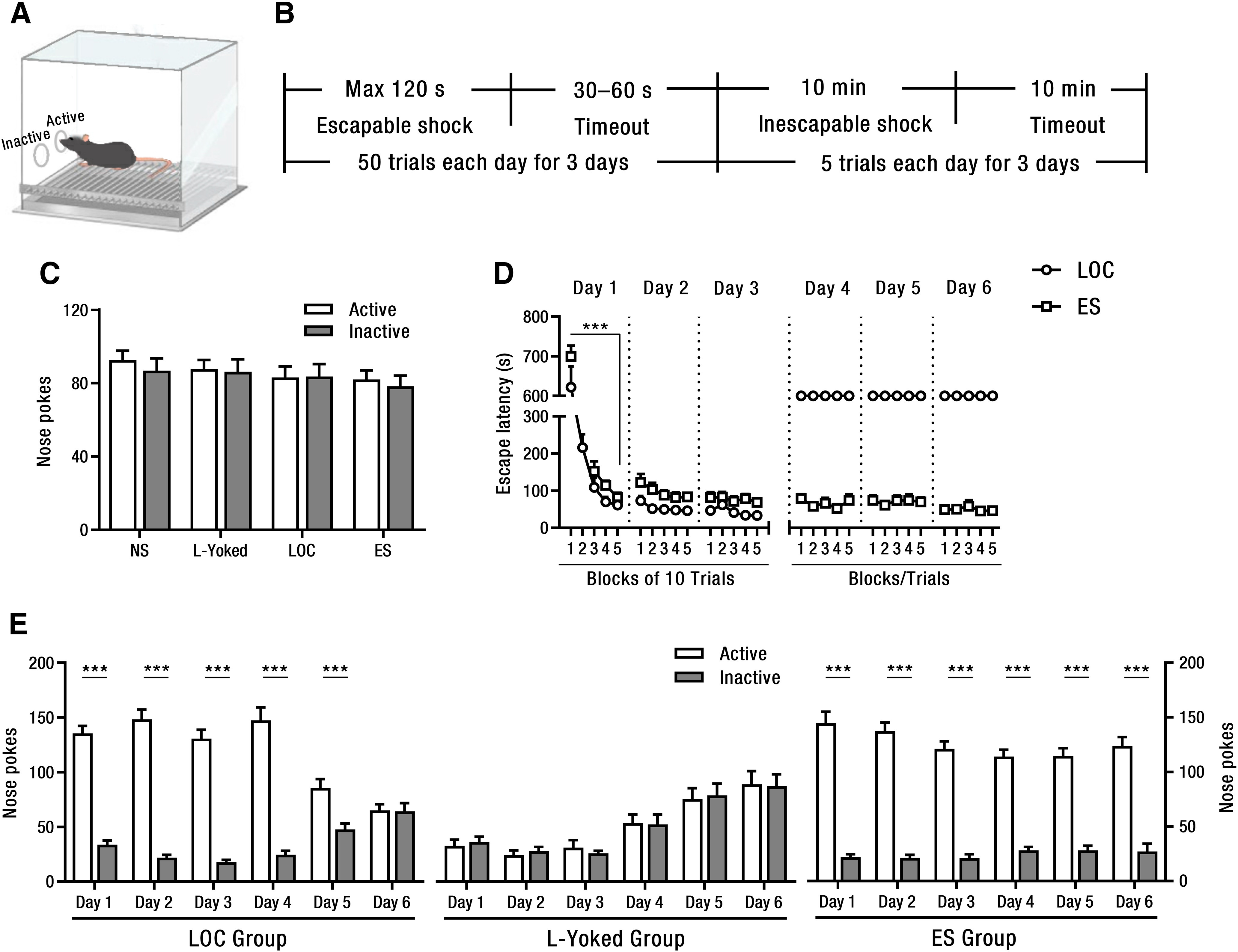
Behavioral results during exposure to escapable shock and inescapable shock. ***A***, A schematic of conditioning operant chamber. Mice terminated footshock after nose poking through the active, but not inactive, nosepoker. ***B***, Procedures of escapable shock and inescapable shock in the LOC group. ***C***, The number of active and inactive nose pokes during acclimation for 100 min (NS: *n* = 41, L-Yoked: *n* = 30, LOC: *n* = 30, ES: *n* = 18). ***D***, Average escape latencies from day 1 to day 6 in the LOC and ES groups (LOC: *n* = 30, ES: *n* = 18). LOC and L-Yoked groups received the identical shock. ***E***, The number of active and inactive nose pokes from day 1 to day 6 in the LOC, L-Yoked, and ES groups (LOC: *n* = 30, L-Yoked: *n* = 30, ES: *n* = 18); ***p <* *0.001. Error bars represent SEM. NS: no shock; ES: escapable shock; LOC: loss of control over shock; L-Yoked: yoked to LOC.

LOC paradigm was performed exactly as described previously (Yao et al., 2019). Mice were introduced to the conditioning operant chambers [30 × 24 × 30 (L × W × H) in cm; MED-Associates] with two nose-poke detectors mounted 2 cm above the metal grid floor for seven consecutive days (Fig. 1*A*). One detector was randomly designated as “active” which was illuminated with a light (20 lux) during shock and its activation triggered shock termination, while the other had no programmed consequence. Twenty-four hours before modeling LOC, mice were allowed to freely explore the chambers for 100 min with NS and randomly divided into LOC, L-Yoked, ES, and NS groups. On days 1–3, LOC mice were exposed to negative reinforcement learning to escape mild footshocks (0.15 mA), while L-Yoked mice were yoked to receive the identical footshocks with the same duration, intensity, and temporal pattern. The procedure consisted of 50 trials each day with a pseudorandom intertrial interval ranging from 30 to 60 s. Shock began simultaneously for both mice in a pair and terminated for both whenever the LOC mice poked the active nosepoker, following a 1.5-s tone (2.9 kHz, 65 dB). If LOC mice did not poke the active nosepoker in 120 s, the shock was automatically terminated. Total shock duration is ∼20 min, the exact time of shock depending on the ability of LOC mice to learn and to respond properly. On days 4–6, LOC mice were exposed to inescapable mild footshocks for the extinction of the learned escape behavior. LOC mice were no longer able to turn off the shock (0.15 mA) by poking the previously active poker and lost control over shocks, while the L-Yoked mice received the identical shocks. The procedure consisted of five trials each day with an intertrial interval of 10 min (Fig. 1*B*). Each trial lasted for 10 min, and the total shock duration was 50 min in each day. ES mice were always exposed to negative reinforcement learning to escape mild footshocks from day 1 to day 6. Mice of the NS group were placed in the operant chambers for the same duration each day but received no shock.

### Shuttle box test

Twenty-four hours after the last exposure to the footshock procedure, escape behavior was observed in shuttle box (Shanghai Jiliang Software Technology Co, Ltd.) using procedures as described previously (Amat et al., 2008; Yao et al., 2019). Mice were placed into the shuttle boxes to freely explore 10 min. This was followed by two footshocks (0.15 mA) at a 60 s interval that could be terminated by shuttling to the other compartment in the shuttle box. These two FR1 trials were followed by a 20-min safety period. This safety period was followed by three FR1 trials and then 25 fixed ratio 2 (FR2) trials. In the FR2 trials, mice needed to shuttle to the other compartment and then back to terminate the shock in the shuttle box, and escape latency was recorded. The maximum duration of one shock was 30 s. If the required responses were not finished, and a 30-s latency was assigned. Total duration of testing was ∼26–35 min.

### SPT

Four to six hours after exposure to LOC model, mice were placed individually in a chamber (22 × 17 × 19 cm, AniLab Software & Instrument Co, Ltd.) equipped with two contact lickometers connected to two bottles. In the adaptation for 2 h, two bottles were filled with normal tap water. A logic electronic circuit was used to detect mice’s licking of the lickometer, which relayed digital “lick” signals to a computer. All mice were placed back in their home cage drinking freely until a 12-h water deprivation (8 P.M. to 8 A.M.). In the preference test lasting for 2 h, the two bottles were filled with 1% sucrose solution and tap water, respectively. Number of licks for tap water and sucrose solution were recorded. The preference index was calculated as the ratio of the number of licking for the sucrose solution to the total number of licking.

### Forced swimming test (FST)

After the SPT, mice were placed in their home cage for 3 h. Then, they were individually placed in a transparent cylinder (18 cm in height, 10 cm in diameter) filled with water (24 ± 1°C, 15-cm height) for 6 min. Their behaviors were observed and recorded by video camera placed on the side of cylinders. The videos were offline analyzed by EthoVision software (EthoVision 10.0 Noldus). Immobility behavior is defined as the lack of all movement except that which was necessary to keep the head above water. Immobile time was scored during the last 5 min.

### ELISAs of serum corticosterone

To determine serum corticosterone concentrations in different sessions of LOC model, blood samples were collected on days 1, 4, 6, and 7 after the shuttle box test between 10 and 11 A.M.. 2 min after stress exposure, mice were anesthetized with isoflurane and a retro-orbital blood sample was taken. After allowing 3 h for clotting to occur, samples were centrifuged at 4°C (4000 rpm, 15 min). The supernatant was collected and frozen at −80°C until the assay was performed for not longer than two months. Serum corticosterone concentrations were determined using the Corticosterone ELISA kit (#ADI-901-097, Enzo Life Sciences) according to the manufacturer’s instructions.

### *In vivo* electrophysiology and analysis

All recordings were conducted blinding to the experimental groups until after the analysis of spontaneous activity; 24−48 h after the shuttle box test, mice were anaesthetized with 20% urethane (1 g/kg, i.p.) placed in a stereotaxic apparatus. Body temperature was maintained at 37 ± 0.5°C. Following craniotomy, a glass microelectrode (2.0 mm o.d. × 1.16 mm i.d.; Harvard Apparatus; 10–20 MΩ) filled with 2 m sodium chloride was directed stereotaxically to the SNc (anterior/posterior, –2.9/–3.5; medial/lateral, ±0.7/1.2; and dorsoventral, –4.0/–4.8). Extracellular signals were amplified (1000×) and filtered (0.3- to 1-kHz bandpass) using a Neurolog system (Digitimer, Ltd.), acquired on-line via a Micro1401 interface and Spike2 software (Cambridge Electronic Design) stored in a computer for off-line analysis.

Neurons identified to be putative DA neurons using the following standardized electrophysiological criteria (Cao et al., 2010; Ungless and Grace, 2012) and anatomic location in SNc were included for off-line analysis in this study: (1) a typical triphasic action potential; (2) an action potential duration from start to negative trough ≥1.1 ms; and (3) a slow firing rate (<10 Hz) with an irregular single spiking pattern. Burst firing identified during analysis which met the following two criteria: (1) onset was defined by two consecutive spikes within an interval <80 ms; and (2) offset with an interval >160 ms. Each data were analyzed at least 5 min of a stable recording with regard to the following parameters: (1) average firing rates; (2) percentage of bursting firing cells (number of burst firing cells/number of total recorded cells × 100%); (3) frequency of bursting; (4) percentages of spikes within bursts (number of spikes within bursts/total number of spikes × 100%; Cao et al., 2010).

### *In vitro* electrophysiology and analysis

Brain slices of SNc were prepared as described previously; 24−48 h after stress exposure, mice were anesthetized with 20% urethane (1 g/kg, i.p.). The brain was removed quickly, and acute brain slices (300 μm) containing the SNc were cut using a microslicer in heated (34°C) artificial CSF (aCSF; 125 mm NaCl, 2.5 mm KCl, 1.25 mm NaH_2_PO_4_, 1 mm MgCl_2_, 2 mm CaCl_2_, 25 mm NaHCO_3_, and 25 mm glucose, pH 7.4, 295–305 mOsm) and saturated by 95% O_2_ and 5% CO_2_. Slices were immediately transferred to a heated (34°C) recovery chamber filled with oxygenated aCSF solution for 1 h. Slices were transferred into a recording chamber at room temperature fitted with a constant flow rate of oxygenated aCSF (2.5 ml/min) containing 20 μmol/l DNQX (Sigma), 50 μmol/l AP (Sigma) and 100 μmol/l picrotoxin (Sigma). Patch pipettes (2–6 MΩ) were filled with an internal solution containing the following: 120 mm potassium gluconate, 20 mm KCl, 2 mm MgCl_2_, 10 mm phosphocreatine, 10 mm HEPES, 2 mm sodium ATP, and 0.4 mm sodium GTP (pH 7.2, 280–290 mOsm). SNc DA neurons were identified by its location using infrared differential interference contrast microscopy (Olympus) and the presence of a hyperpolarization-activated cation (I_h_), which a typical criterion to determine DA neurons (Saal et al., 2003). I_h_ currents were measured in whole-cell voltage-clamp mode by repetitive 2-s pulses with 10-mV incremental steps from a holding potential of –60 to –130 mV. Cell excitability was measured in current-clamp mode with 2-s incremental steps of current injections (0, 25, 50, 75, 100, 125, 150, 175, and 200 pA). Series resistance was monitored during all recording. Signals were amplified using a Multiclamp 700B, digitized with Digidata 1550, and acquired with pCLAMP 10 (Molecular devices). Data were offline analyzed using Clampfit.

### Surgery and virus injection

AAV2/9-Ef1α-DIO-GCaMP6m-WPRE-hGH pA and AAV2/9-Ef1α-DIO-eYFP-WPRE-hGH pA virus plasmids (titers 1–5 × 10^12^ viral particles per ml) were purchased from BrainVTA Co, Ltd., Wuhan (Zhang et al., 2017). DAT-Cre mice were anesthetized with isoflurane and head-fixed to a stereotaxic apparatus (RWD Life Science). Ophthalmic ointment was applied to prevent eyes from drying. After making a small craniotomy, viral vectors (200 nl) were slowly injected (40 nl/min) into the SNc (anterior/posterior, +3.1 mm; medial/lateral, +1 mm; dorsal/ventral, –4.2 mm) using a glass pipette controlled by a microsyringe pump (KD Scientific). The glass pipette was slowly withdrawn after stopping 10 min. Following virus injection, an optical fiber [200-μm core diameter, 0.37 numerical aperture (NA); Shanghai Fiblaser] was placed in a ceramic ferrule, implanted into the SNc and secured to the skull with dental cement. After surgery, each two mice were housed in a cage and allowed to recover at least two weeks. The placements of implantable optical fibers were confirmed after behavioral tests in all animals.

### Fiber photometry and data analysis

Fiber photometry was used to record the population activity of neurons expressing a genetically encoded Ca^2+^ indicator in cell bodies of awake mice (Gunaydin et al., 2014; Zhong et al., 2017). The signals were record by the multi-channel fiber photometry recording system (Thinkertech; Guo et al., 2015; Zhong et al., 2017). An optical fiber (200-μm core diameter, 0.37 NA; 2 m long) guided the light between the commutator and the implanted optical fiber. The laser intensity was measured at the tip of optical fiber and adjusted to 10–20 μW to minimize photobleaching. The signal was collected and digitalized at 50 Hz by ThorCam-DAQ. The fluorescence change (ΔF/F) values were calculated as (F–F_0_)/F_0_, where F_0_ is the baseline fluorescence signals averaged over a 1.5-s-long control time window (typically set 0.5 s) before the onset of licking sucrose solution. ΔF/F values are presented as average plots with a shaded area indicating the SEM. To analyze the response magnitudes to sucrose solution intake before and after stress exposure, we calculated the area under the curve (AUC) during the 3-s time window following the onset of licking.

### Immunostaining

Mice were anesthetized with pentobarbital and then intracardially perfused with 0.9% saline followed by 4% paraformaldehyde in PBS. After postfixation overnight (4°C), the mouse brain was cryoprotected with 30% sucrose and then sectioned coronally (16 μm in thickness) with a microtome (Leica CM1900). For immunohistochemistry, the sections were blocked with 3% BSA in PBS with 0.3% Triton X-100 and incubated with a rabbit polyclonal antibody against tyrosine hydroxylase (TH; 1:200; Abcam, #ab112) at 4°C overnight. After washing with PBS, the brain sections were incubated with Alexa Fluor 594 Affinipure Goat anti-rabbit IgG (1:200; Yeasen) for 2 h at room temperature. PBS-washed sections were then coverslipped with 50% glycerol mounting medium. The stained sections were imaged with a microscope (Axio Imager M2, Zeiss).

### Experimental design and statistical analysis

Mice were excluded from statistical analysis when they learned to use various gestures or actions to avoid shock without nose poking. The numbers of nosepokes were analyzed using unpaired two-tailed Student’s *t* test, respectively. Escape latency was analyzed using one-way ANOVA followed by a Turkey’s *post hoc* test. *In vivo* recording and serum corticosterone concentration data were analyzed using one-way ANOVA followed by a Tukey’s *post hoc* test. The changes of percentage of burst firing cells were analyzed using χ^2^ test. I_h_ currents and excitability were analyzed using a two-way repeated measures ANOVA followed by a Tukey’s *post hoc* test when appropriate, with group as between subject factor, and injections current and voltage as within subject factor, respectively. AUC was analyzed using a two-way repeated measures ANOVA with group as between subject factor and test session as within subject factor. At each group (NS, ES, L-Yoked, and LOC), comparison was analyzed with paired two-tailed Student’s *t* test. Permutation test was used to analyze the statistical significance of the fluorescence change from the baseline of fluorescence on licking onset (Groppe et al., 2011; Li et al., 2016). Α level was set as 0.001 to compare the ΔF/F values at each point with the fluorescence baseline. Each point above the 99.9% fraction of baseline ΔF/F values were considered significant increase. Red color was superimposed on the average fluorescence curve indicating significant increase; *p *<* *0.05 was considered statistically significant. SPSS 20.0 was used to perform statistical analysis. Figures were generated using Prism 7 (GraphPad) and MATLAB software.

## Results

### Mice in the LOC group acquired and then lost control over footshocks

During the freely exploring period before the stress procedure, there were no significant differences in the numbers of the active and the inactive nosepokes (Fig. 1*C*), ruling out the preference between the two nosepokers. And then, during the negative reinforcement learning in both the LOC and the ES groups, the escape latency decreased rapidly across the five stimulation blocks on day 1 (LOC: *F*_(4,145)_ = 54.81, *p *<* *0.001, ES: *F*_(4,855)_ = 88.3, *p *<* *0.001; Fig. 1*D*). Meanwhile, the number of the active nosepoke in both the LOC (day 1: *t*_(58)_ = 12.98, *p *<* *0.001; day 2: *t*_(58)_ = 13.69, *p *<* *0.001; day 3: *t*_(58)_ = 13.42, *p *<* *0.001) and the ES (day 1: *t*_(34)_ = 11.18, *p *<* *0.001; day 2: *t*_(34)_ = 13.69, *p *<* *0.001; day 3: *t*_(34)_ = 13.01, *p *<* *0.001) groups was significantly larger than that of the inactive one on days 1–3, indicating that mice in both the LOC and the ES groups learned to terminate the shock by poking the active nosepoker and acquired control over the stressor. In contrast, there was no difference between the numbers of the active and inactive nosepoke in the L-Yoked group. On days 4–6, the footshock was no longer able to be terminated by poking the previously active poker in LOC mice. The number of the active nosepoke was still significantly larger than that of the inactive in the LOC group on days 4–5 (day 4: *t*_(58)_ = 9.85, *p *<* *0.001; day 5: *t*_(58)_ = 3.95, *p *<* *0.001), but this difference was absent in day 6 (*t*_(58)_ = 0.09, *p *=* *0.93; Fig. 1*E*), indicating an extinction of the learned escape behaviors in the LOC group. The number of the active nosepoke was always significantly larger than that of the inactive in the ES group on days 4–6 (day 4: *t*_(34)_ = 12.33, *p *<* *0.001; day 5: *t*_(34)_ = 10.44, *p *<* *0.001; day 6: *t*_(34)_ = 9.05, *p *<* *0.001), indicating that ES mice were always able to control the termination of shock. Again, no differences between the numbers of the two nosepokers were observed in the L-Yoked group on days 4–6 (Fig. 1*E*). Subsequently, depression-like behaviors were assessed by SPT and FST. The results were consistent with the previous findings that the LOC mice exhibited a significant reduction in appetite to natural reward and a more serious despair-like behavior, but the ES mice did not exhibit the similar reduction and behavioral deficit (Yao et al., 2019; Extended Data Fig. 1-1).

10.1523/ENEURO.0044-21.2021.f1-1 Extended Data Figure 1-1 LOC over shock produced significant depression-like behaviors. ***A***, In the SPT, the LOC group exhibited a significant reduction in sucrose preference index, as compared with the other three groups. Preference index is calculated as the ratio of the number of licks for the sucrose solution to the total number of licks. ***B***, In the FST, both the LOC and the L-Yoked groups exhibited significantly longer immobile time than the ES and the NS groups, respectively. In addition, the LOC group showed even longer immobile time than the L-Yoked group; *p <* *0.05, **p <* *0.01, ***p <* *0.001. Error bars represent SEM. LOC: loss of control over shock; L-Yoked: yoked to LOC; ES: escape shock; NS: no shock. NS: *n* = 8, L-Yoked: *n* = 8, LOC: *n* = 8, ES: *n* = 6. Download Figure 1-1, TIF file.

### Both lack and LOC over shock increase the spontaneous activity of putative SNc DA neurons

**Figure 2.**
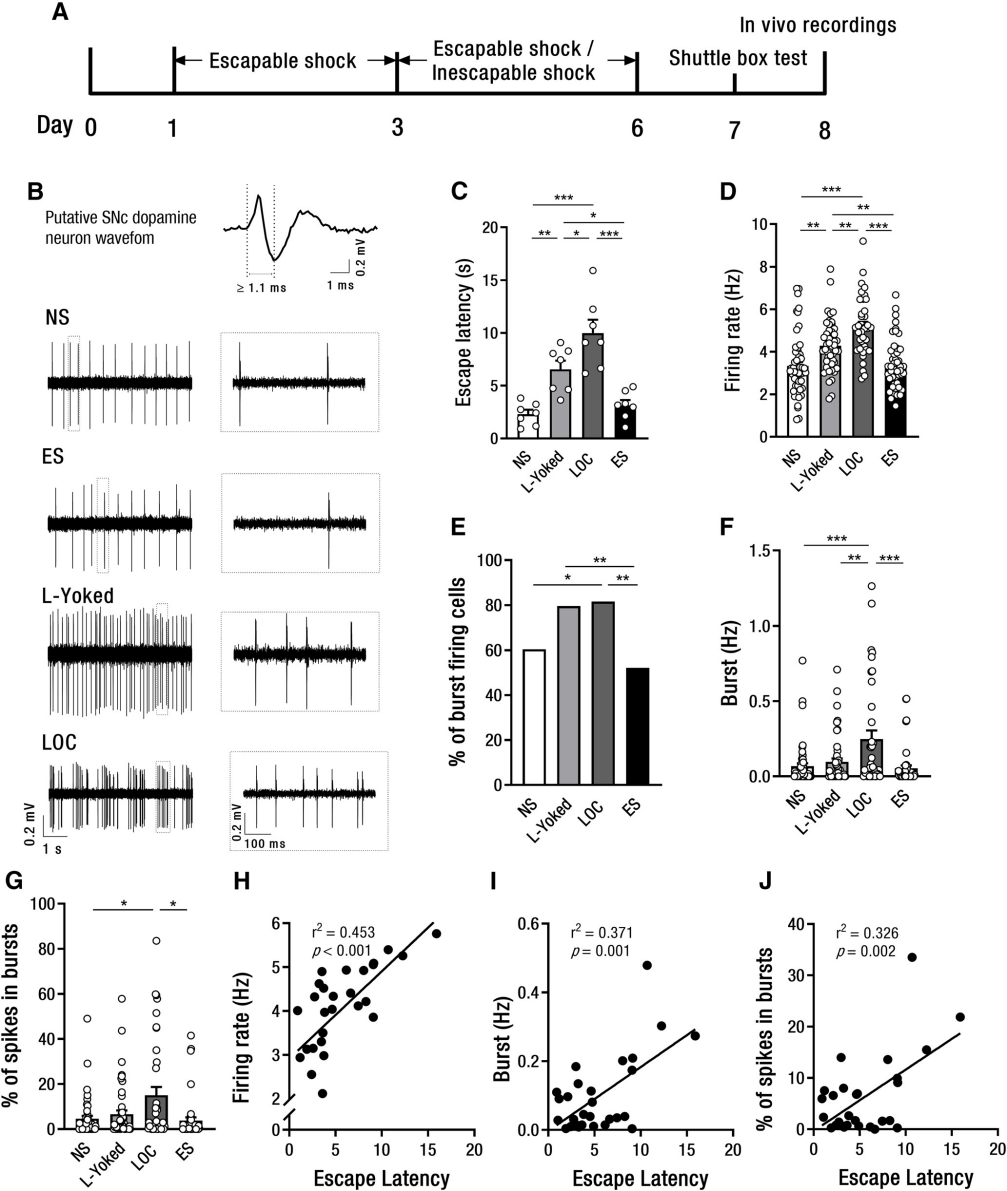
Both lack and loss of control over shock increase the activity of SNc DA neurons. ***A***, Timeline of LOC model, shuttle box test, and *in vivo* recordings. ***B***, Representative *in vivo* SNc DA neuron firing traces for the NS, ES, L-Yoked, and LOC mice. ***C***, Mean escape latencies of 25 FR2 escape trials in the shuttle box test on day 7 (*n* = 7 per group). ***D***, SNc DA neuron firing rates in the LOC, L-Yoked, ES, and NS mice. ***E***, SNc DA neuron percentage of bursting firing cells among the four groups. ***F***, SNc DA neuron frequency of busting among the four groups. ***G***, SNc DA neuron percentage of spikes in bursts among the four groups. ***H***, Correlation between average SNc DA neuron firing rates and mean escape latencies in the shuttle box test in all four groups (*n* = 28 mice). ***I***, Correlation between average SNc DA neuron frequency of bursting and mean escape latencies in the shuttle box test in all four groups (*n* = 28 mice). ***J***, Correlation between average SNc DA neuron percentage of spikes in bursts and mean escape latencies in the shuttle box test in all four groups (*n* = 28 mice); *p <* *0.05, **p <* *0.01, ***p <* *0.001. Error bars represent SEM. NS: no shock; ES: escapable shock; LOC: loss of control over shock; L-Yoked: yoked to LOC. NS: *n* = 53 cells from 7 mice; ES: *n* = 46 cells from 7 mice; L-Yoked: *n* = 49 cells from 7 mice; LOC: *n* = 38 cells from 7 mice.

The spontaneous unit firing activity of SNc DA neurons in anesthetized mice of the LOC, L-Yoked, ES, and NS groups was recorded 24 h after the shuttle box test following the stress procedure (Fig. 2*A*,*B*). There was a significant difference in escape latency (*F*_(3,24)_ = 18.25, *p *<* *0.001; Fig. 2*C*) and firing rates among the four groups (*F*_(3,182)_ = 19.06, *p *<* *0.001; Fig. 2*D*). The escape latency was significantly longer in the LOC group than that in the L-Yoked group (*p *=* *0.033), the ES group (*p *<* *0.001), and the NS group (*p *<* *0.001), respectively. The escape latency in the L-Yoked group was also significantly longer than that in the ES (*p *=* *0.034) and the NS (*p *=* *0.006) groups, respectively. There was no significant difference in the escape latency between the ES and the NS groups. This result is consistent with our previous finding that LOC over stressor produces more serious escape deficits and helplessness, while control over shock prevents the deleterious effects of shock per se (Maier and Seligman, 2016; Yao et al., 2019). Meanwhile, the average firing rate in the LOC group was significantly larger than that in the L-Yoked (*p *=* *0.009), the ES (*p *<* *0.001), and the NS (*p *<* *0.001) groups, respectively. The average firing rate in the L-Yoked group was also significantly larger than that the ES group (*p *=* *0.008) and the NS group (*p *=* *0.001), respectively. There was no significant difference in the average firing rate between the ES and the NS groups. These results indicate that both lack and loss of control over shock enhance the basal activity of putative SNc DA neurons, and loss of control produces even more significant enhancement, but this enhancement is eliminated by acquiring control over shock.

The activity of DA neurons is known to occur either in single-spike or in burst firing mode. The bursting is usually involved in the signaling for salient or novel events (Yadid and Friedman, 2008). We further found that the firing pattern of SNc DA neurons was significantly altered by shock controllability; 60.38% (32/53) of SNc DA neurons recorded from NS mice showed at least one burst-firing event in a 5 min recording period. This percentage of bursting cells increased to 81.58% (31/38) in LOC mice and 79.59% (39/49) in L-Yoked mice (χ^2^ = 12.9, *p *=* *0.005; Fig. 2*E*), but showed no increase in ES mice (52.17%, 24/46). Furthermore, LOC mice exhibited significantly higher frequency of bursting (*F*_(3,182)_ = 7.83, *p *<* *0.001; Fig. 2*F*) and the percentage of spikes in bursts (*F*_(3,182)_ = 3.75, *p *=* *0.012; Fig. 2*G*) in putative SNc DA neurons when compared with ES and NS mice, indicating that loss of control over shock enhances the burst activity of SNc DA neurons.

There was a significant positive correlation between the average firing rate of putative SNc DA neurons and the escape latency of the mice in which the DA neurons were recorded (*r*^2^ = 0.45, *p *<* *0.001; Fig. 2*H*). Consistently, the frequency of bursting (*r*^2^ = 0.37, *p *=* *0.001; Fig. 2*I*) and the percentage of spikes in bursts (*r*^2^ = 0.33, *p *=* *0.002; Fig. 2*J*) of the neurons from each mouse was also positively correlated with the escape latency, suggesting that the increased firing rates were associated with the escape deficits produced by stress exposure.

### Dynamic changes in the level of serum corticosterone in the stress exposure

**Figure 3.**
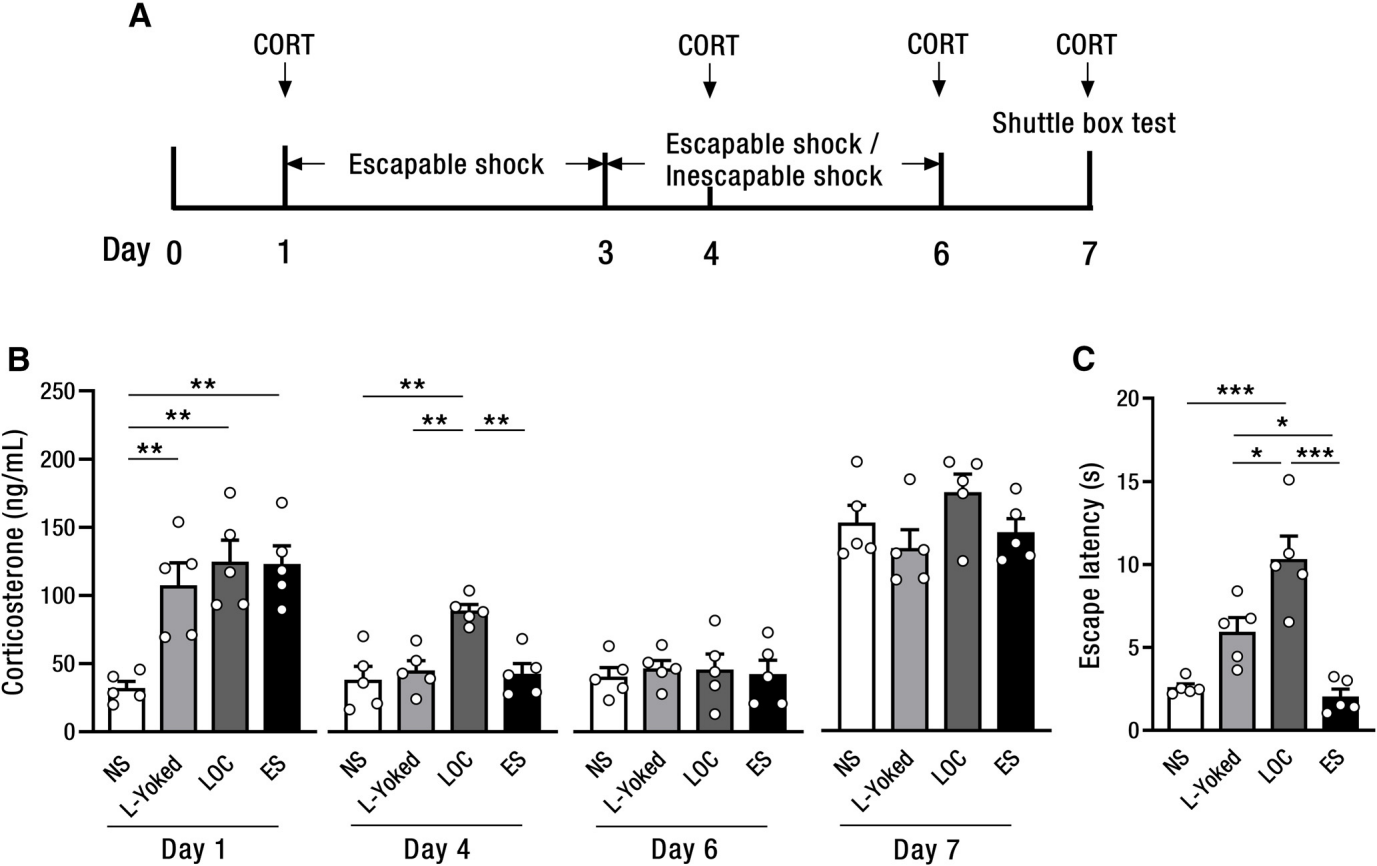
Dynamic changes in the level of serum corticosterone following the stress exposure. ***A***, Timeline of stress exposure, shuttle box test, and taking blood to determine serum corticosterone concentrations. ***B***, The levels of serum corticosterone 2 min after stress exposure at various time (days 1, 4, 6, and 7 after the shuttle box test). LOC mice were exposed to escapable shocks on days 1–3, and then to inescapable shocks on days 4–6, with L-Yoked mice receiving the identical inescapable shocks. ES mice were exposed to escapable shocks on days 1–6. NS mice were placed in the same environment but receiving no shock. On day 7, the four groups were exposed to shuttle box test. ***C***, Mean escape latencies of 25 FR2 escape trials in shuttle box test; *p <* *0.05, **p <* *0.01, ***p <* *0.001. Error bars represent SEM. CORT: corticosterone. LOC: loss of control over shock; L-Yoked: yoked to LOC; ES: escapable shock; NS: no shock. *n* = 5 per group.

In order to investigate the release of stress-related hormones in response to different stress exposure, mice of the LOC, L-Yoked, ES, and NS groups exposed to stress procedures and then randomly divided into four cohorts to determine the levels of serum corticosterone on day 1, day 4, day 6, and day 7 after the shuttle box test, respectively (Fig. 3*A*). The results show that there was a significant difference in the levels of serum corticosterone among the four groups on day 1 (*F*_(3,16)_ = 10.81, *p *<* *0.001; Fig. 3*B*). The levels of serum corticosterone in the LOC (*p *=* *0.001), the ES (*p *=* *0.001), and the L-Yoked (*p *=* *0.005) groups were all significantly higher than that in the NS group, but there was no significant difference among the LOC, the ES, and the L-Yoked groups, suggesting that stress exposure led to an elevation of serum corticosterone level, and this elevation was independent of stress controllability. However, the levels of serum corticosterone in the LOC group were significantly higher, respectively, than that in the L-Yoked, the ES, and the NS groups on day 4 (*F*_(3,16)_ = 10.14, *p *=* *0.001), showing that when the controllable shock became uncontrollable, the levels of serum corticosterone increased significantly. In contrast, no significant difference was observed among the four groups on day 6 (Fig. 3*B*), suggesting that there might be some adaptations in corticosterone release after prolonged exposure to uncontrollable shock.

To further investigate the release of corticosterone in response to shuttle box test following stress exposure among the four groups, serum corticosterone levels were determined on day 7 after the shuttle box test. The results show that there was a significant difference in the escape latency in the shuttle box test (*F*_(3,16)_ = 20.12, *p *<* *0.001; Fig. 3*C*). Tukey’s *post hoc* test showed that the escape latency in the LOC group was significantly longer than that in the L-Yoked (*p *=* *0.01), the ES (*p *<* *0.001), and the NS groups (*p *<* *0.001), respectively. The escape latency in the L-Yoked group was also significantly longer than that in the ES group (*p *=* *0.02). This result is consistent with the previous findings that LOC over shock produces more serious escape deficit (Yao et al., 2019). Meanwhile, there was no significant difference in the levels of serum corticosterone among the four groups on day 7 after the shuttle box test (*F*_(3,16)_ = 2.05, *p *=* *0.15, Fig. 3*B*), indicating that escape behavioral differences in shuttle box are not caused by the release of corticosterone.

### Both lack and LOC over shock increase intrinsic excitability of SNc DA neurons

**Figure 4.**
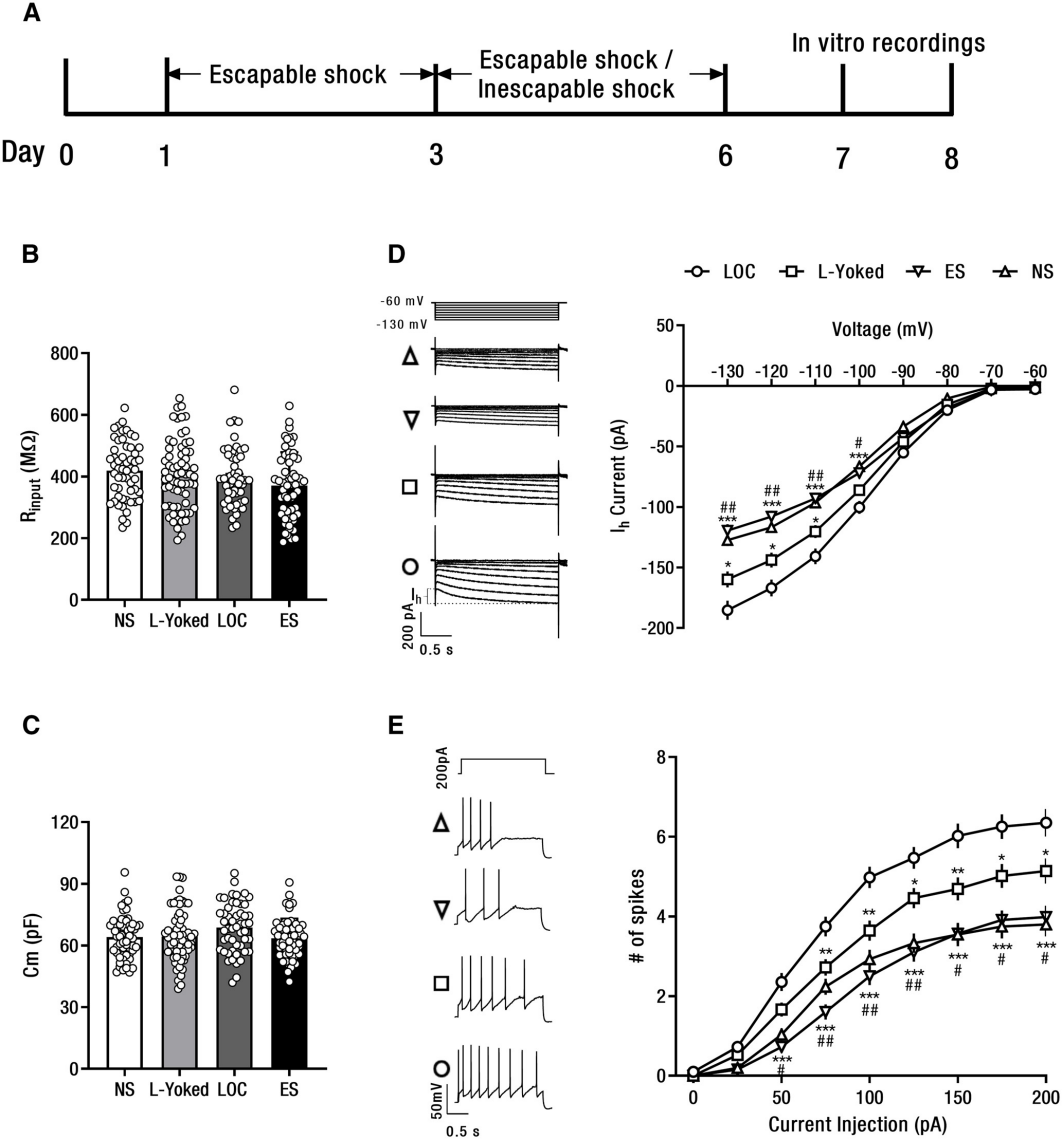
Both lack and loss of control over shock increase the excitability of SNc DA neurons. ***A***, Timeline of LOC model and in vitro recordings. ***B***, Input membrane resistance (R_input_) of SNc DA neurons. ***C***, Membrane capacitance (Cm) of SNc DA neurons. ***D***, Representative I_h_ traces (left) and I-V graph of I_h_ (right). ***E***, Representative spike traces after current injection (left, 200 pA) and the number of spikes elicited to current injections of SNc DA neurons from the LOC, L-Yoked, and NS mice; *p <* *0.05, **p <* *0.01, ***p <* *0.001 versus LOC group; #*p *<* *0.05, ##*p *<* *0.01 versus L-Yoked group. Error bars represent SEM. NS: no shock; ES: escapable shock; LOC: loss of control over shock; L-Yoked: yoked to LOC. NS: *n* = 55 cells from 6 mice; ES: *n* = 58 cells from 6 mice; L-Yoked: *n* = 65 cells from 6 mice; LOC: *n* = 51 from 6 mice.

Previous study has discovered excitatory hyperpolarization-activated currents (I_h_) expressed in DA neurons can intrinsically modulate neuronal firing that may contribute to the transition from single spike to burst mode (Cao et al., 2010). To investigate the intrinsic electrophysiological properties of SNc DA neurons after stress exposure (Fig. 4*A*), whole-cell voltage-clamp recordings were used to measure excitatory I_h_ current from DA neurons in SNc slice preparations of the LOC, L-Yoked, ES, and NS groups. DA neurons were identified electrophysiologically by the presence of a large I_h_ current (Saal et al., 2003). The results show that there were no significant differences in the input membrane resistance and membrane capacitance of SNc DA neurons recorded from the LOC, L-Yoked, ES, and NS mice (Fig. 4*B*,*C*). However, the main effects of group (*F*_(3,225)_ = 16.93, *p *<* *0.001) and clamp voltage (*F*_(7,1575)_ = 1969.56, *p *<* *0.001), and the interaction between group and clamp voltage (*F*_(21,1575)_ = 20.38, *p *<* *0.001) were significant (Fig. 4*D*). I_h_ current in SNc DA neurons in the LOC group was significantly larger than that in the L-Yoked, the ES, and the NS groups, respectively. In addition, I_h_ current in the L-Yoked group was significantly larger than that in the ES and the NS groups, respectively, showing that both lack and loss of control over shock enhance the I_h_ current in SNc DA neurons, and loss of control over shock produces a significantly larger enhancement. There was no significant difference in I_h_ current amplitude between the ES and the NS groups, suggesting the prevention of I_h_ current enhancement by acquiring control over shock.

To further examine the above results, intrinsic excitability among the four groups was investigated in current-clamp modes. Repeated measure ANOVA revealed significant main effects of group (*F*_(3,225)_ = 18.92, *p *<* *0.001) and current injection (*F*_(8,1800_ = 684.54, *p *<* *0.001), and the interaction between group and current injection (*F*_(24,1800)_ = 8.91, *p *<* *0.001; Fig. 4*E*). SNc DA neurons in the LOC group generated significantly more action potentials in response to current injection than that in the L-Yoked, the ES, and the NS groups, respectively. DA neurons in the L-Yoked group also generated significantly more action potentials to current injection than that in the ES and the NS groups, respectively. Again, there was no significant difference in action potential numbers in response to current injections between the ES and the NS groups, suggesting prevention of excitability enhancement by acquiring control over shock.

### Loss of control over shock decreases the natural reward-induced calcium signal of SNc DA neurons

**Figure 5.**
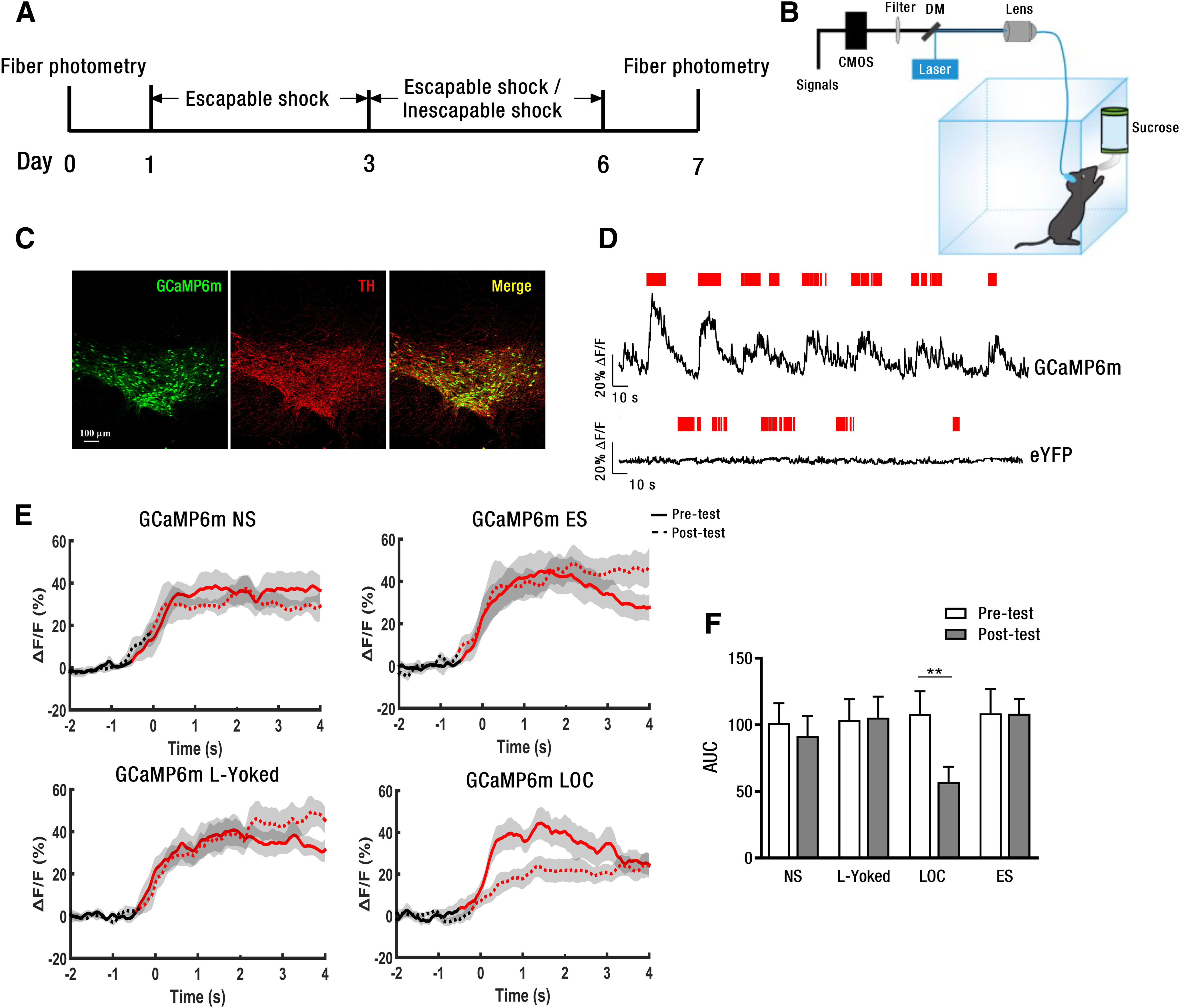
Loss of control over shock decreases the natural reward-induced calcium signal of SNc DA neurons. ***A***, Timeline of LOC model and fiber photometry. ***B***, Fiber photometry of calcium signal from freely behaving mice voluntarily licking sucrose solution. ***C***, Expression of GCaMP6m (green) in TH-immunopositive neurons (red) in the SNc of a DAT-Cre mouse. ***D***, Raw traces from expressing GCaMP6m (top) and eYFP (bottom) in the SNc of the DAT-Cre mice during the sucrose solution intake. GCaMP6m fluorescence showed a robust increase during the sucrose licking epochs (res dashes). ***E***, Peri-event plots of average calcium signals during licking sucrose solution before and after stress exposure in the NS, ES, L-Yoked, and LOC groups, respectively. Red color superimposed on the black solid and dashed line indicate significant increases from baseline (*p *<* *0.001). ***F***, Quantification of SNc fluorescence evoked by licking sucrose solution before and after stress exposure in the NS, L-Yoked, LOC, and ES groups (**p <* *0.01). AUC, area under curve. Error bars represent SEM. NS: no shock; LOC: loss of control over shock; L-Yoked: yoked to LOC; ES: escapable shock. NS: *n* = 8, L-Yoked: *n* = 7, LOC: *n* = 7, ES: *n* = 7.

Our above *in vivo* and in vitro electrophysiological data demonstrated that LOC over shock significantly enhances the basal activity and intrinsic excitability of SNc DA neurons. Thus, to address whether this enhancement of basal activity alters the responses of SNc DA neurons to natural reward, we used fiber photometry to investigate the changes of SNc DA neuronal activity when the mice were voluntarily consuming sucrose solutions (Fig. 5*A*,*B*). There was reliable and efficient expression of GCaMP6m in neurons expressing TH, the rate-limiting enzyme for DA neurons in the brain (Fig. 5*C*). When mice licked for sucrose solution, the fluorescence in SNc GCaMP6m showed a robust increase, but this effect was absent in control mice expressing eYFP (Fig. 5*D*).

We next applied fiber photometry to record the calcium signal in SNc DA neurons of mice expressing GCaMP6m given access to sucrose solution before and after stress exposure in the four groups. We observed a significant increase in SNc GCaMP6m fluorescence signal during licking sucrose solution in the LOC, the L-Yoked, the ES, and the NS groups (Fig. 5*E*). We then calculated the AUC during licking sucrose solution for the first 3 s and found a significant main effect of session (*F*_(1,25)_ = 7.04, *p *=* *0.01) and the interaction between group and session (*F*_(3,25)_ = 4.73, *p *=* *0.009; Fig. 5*F*). Calcium response in SNc DA neurons in the mice of LOC group licking sucrose solution after stress-exposure showed a significant decrease compared with the response before stress-exposure (*p *=* *0.003), but no significant differences were observed in the L-Yoked, the ES, or the NS mice, suggesting that loss of control over shock produces a significant decrease of natural reward-induced calcium signal in SNc DA neurons.

## Discussion

Prolonged stress produces neural maladaptations in midbrain dopaminergic system and thereby leads to express of emotional and behavioral disorders (Hollon et al., 2015). In the present study, mice of the L-Yoked and the LOC groups were exposed to identical mild footshock for 6 d, during which to the L-Yoked group the stress was uncontrollable but to the LOC it was firstly controllable and then uncontrollable, forming a defeat experience of the LOC mice. The results show that SNc DA neurons in both the L-Yoked and the LOC groups exhibit enhanced basal firing activities, and this enhancement is significantly larger in the LOC group than in the L-Yoked group. The enhancement in SNc DA neuron activity exhibits a positive correlation to the escape deficits induced by the stress exposure. These results suggest that behavioral deficits induced by LOC over stress increase the firing activity of DA neurons in SNc. As well known, SNc DA neurons play key roles in motor functions, and their degeneration results in movement deficits (Surmeier, 2018). Therefore, the alterations in SNc DA neuron activity imply functional differences in motor learning behaviors.

Previous literatures have reported that unpredictable mild stress reduces but social defeat stress increases the firing activity of midbrain DA neurons in VTA (Moore et al., 2001; Cao et al., 2010). However, in the VTA of anesthetized rats, the population of presumed DA neurons that are excited by aversive stimuli is actually not dopaminergic. The identified DA neurons were inhibited by the aversive stimulus (Ungless et al., 2004). On the other hand, some SNc DA neurons are reported to be excited by aversive stimuli (Matsumoto and Hikosaka, 2009; Ungless and Grace, 2012). Diverse inputs to DA neurons and outputs to distinct regions in the SNc and VTA are likely to be important determinants as to how neurons respond to aversive stimuli, and therefore may contribute to their diverse roles in behavior (Collins and Saunders, 2020). Therefore, the distinct activity of DA neurons in the SNc and VTA during different brain states still needs further investigation (Brown et al., 2009; Walczak and Błasiak, 2017). It also seems to be important to further investigate different DA subpopulations in response to distinct stressors that may result in behavioral deficits.

It has long been recognized that stressor activates the hypothalamic-pituitary-adrenal (HPA) axis, releasing stress-related hormones (Joëls and Baram, 2009). In the present study, we found that the level of serum corticosterone changed dynamically during the stress exposure, but was similar in the L-Yoked, the LOC, the ES, and the naive groups after the stress exposure, showing that the enhancement in DA neuronal firing is not acutely caused by corticosterone release. The result is consistent with previous findings showing the dynamic adaptive processes of the HPA axis during persistent and repeated uncontrollable stress (Cole et al., 2000; Reber et al., 2007) and also confirmed the observation in the classic learned helplessness paradigm (Maier and Seligman, 2016), in which changes in controllability showed no significant influence on the long-term activity of the HPA axis.

It is well known that DA neurons displayed two patterns of spontaneous firing activity: a tonic, single spike firing and a phasic, burst firing, which regulate synaptic levels of DA and distinct behavioral function. The burst firing pattern, produced on a timescale of hundreds of milliseconds, plays a key role in encoding salience signals via phasic release of DA, while the single spike firing pattern depends on the spontaneous firing of DA neurons with slower changes in tonic levels of DA (Floresco et al., 2003; Grace et al., 2007). I_h_ not only regulates the spontaneous firing of DA neurons, but also regulates the transition of firing mode between tonic and phasic firing (Cao et al., 2010). Changes in tonic level of DA can directly influence the magnitude of the phasic DA response regulated by bursting firing in behavior (Floresco et al., 2003). Indeed, we also found that I_h_ current was more significantly enhanced in the LOC than in the L-Yoked group. Therefore, the enhanced I_h_ current may contribute to the increased firing of SNc DA neurons induced by both lack and LOC over shock. Most importantly, these results are consistent with previous findings that chronic social defeat leads to the increased firing of DA neurons via modulation of I_h_ current, which emphasizes psychosocial aspects of the stressors, although in the social defeat paradigm it is very difficult to separate physiological and psychological components of defeat-induced depression, and also does not rule out physiological effects (Hammels et al., 2015). The results in the present study indicate that the larger enhancement of basal activity of SNc DA neurons through the modulation of I_h_ current in the LOC group may contribute to psychological stress-related behavioral deficits following LOC over stressors.

Previous studies have shown that when neurons are modulated to be more excitable than normal and exhibit larger spontaneous basal firing activity, they may generate reduced further responses to excitatory inputs (Hollon et al., 2015). Responsive activation of midbrain DA neurons mediate normal social behaviors and responses to natural rewards. In the present and a series of previous studies, mice experienced chronic stress exhibit enhanced DA neuron activity but showed deficits in social interaction and preference to natural rewards (Krishnan et al., 2007). We observed that the natural reward-induced responses in calcium influx of SNc DA neurons decreased significantly in the LOC group. Such deficits in social interaction and response to natural rewards might also be caused by the inability of DA neurons to generate further firing responses. This speculation remains to be clarified in future studies. In addition, the psychological component of stress may also contribute to the reduced responses to reward, which is also a possible mechanism for the reduction of hedonic capacity in clinical depressed patients (Watson et al., 1988; Pizzagalli et al., 2008). We found that loss of control over shock produces more significant enhancement in the basal activity of DA neurons than what caused by the effects of shock per se, and then attenuated their response to natural reward.

## Synthesis

Reviewing Editor: Jonathan Lee, University of Birmingham Decisions are customarily a result of the Reviewing Editor and the peer reviewers coming together and discussing their recommendations until a consensus is reached. When revisions are invited, a fact-based synthesis statement explaining their decision and outlining what is needed to prepare a revision will be listed below. The following reviewer(s) agreed to reveal their identity: NONE. Note: If this manuscript was transferred from JNeurosci and a decision was made to accept the manuscript without peer review, a brief statement to this effect will instead be what is listed below.

There is agreement among the editor and reviewers that the manuscript presents an interesting and novel set of data emerging from well-designed experiments. However, it is difficult to evaluate fully the merits of the study because of the way in which it has been presented.

Most importantly, the literature into which the study is contextualised and interpreted relates exclusively to the effects of chronic stress on VTA dopaminergic neurons. With the current set of data on SNc dopaminergic neurons (and without recording of VTA dopaminergic neurons), there is a substantial and important disconnect between the literature and the data. This needs to be addressed by refocussing the manuscript on the SNc and acknowledging/discussing the differences between the SNc and VTA. In particular, the results should be interpreted in relation to established functions and behaviours linked to the SNc.

A second, related, major comment is that the manuscript has an explicit focus on the comparison between the LOC model and classical stress models. This comparison is somewhat weakened by the absence of additional behavioural measures (or at least robust discussion in relation to previous characterisation of the model). Therefore, the manuscript should be substantially revised either to reduce such a comparative emphasis, or to add more robust discussion. This relates explicitly to the above comment about interpretation of the results in relation primarily to SNc behaviours.

Below are presented the verbatim parts of the reviews pertaining to these issues, as well as some additional comments that should be addressed. Please note that there were several additional more minor concerns raised by each of the reviewers. However, because the manuscript requires major revision at the level of theoretical grounding and interpretation, it would be better for this to be addressed first; additional comments will follow with the review of the revised submission.

--------------------------

Yao et al has great potential. The authors show that extended shock exposure results in deficits in another shock-based test, and this deficit is exacerbated by a loss of control over the shocks. This coincides with extended shock induced increases in SN firing rate rates and bursting phenomena, and ex-vivo enhancements of Ih currents and excitability. In addition authors show circulating corticosterone is not correlated to these outcomes. However, the manuscript suffers from a lack of contextualizing both the paradigm and results.

Major:

• In this study the authors do not show their model results in classical stress induced behaviors, rendering their proclaimed comparisons with chronic stress models difficult to believe. Their claim would be made stronger with additional metrics such as reduced preference for sucrose or social interaction, enhanced risk aversion metrics like elevated plus maze, novelty suppressed feeding, or a light-dark box, rather than another learning-based shuttle-box test.

• The authors do not sufficiently justify or contextualize their results in terms of SN DA neurons. As many of their sources focus on VTA neurons, what implication does this have on the field? Are stress-induced behavioral deficits just failure to coordinate actions?

• The authors do not discuss or hypothesis the relevance of the bursting phenomenon vs increased firing rate for stress-induced behaviors.

• No exclusion criteria are described in the manuscript

-------------------------

In this study, the authors investigate the consequences of various footstock controllability paradigms on the activity of SNC dopaminergic neurons in mice. They correlate electrophysiological results with the ability of different groups of animals to escape further footshocks to (shuttle box test) and with the capacity of SNC DA neurons to get activated (ca2 + imaging) in response to sucrose consumption.

Notably, the authors successfully show that the behavioural (increased escape latency) and electrophysiological consequences (increased tonic and phasic activity of SNC DA neurons) observed in mice having undergone unpredictable footstock for 6 days (L-Yoked mice) were exacerbated in mice which controlled and then lost this control (prediction) of footshocks (LOC mice). The authors also show that these exacerbations of alterations do not correlate with an increase in blood corticosterone levels.

The experimental methods are well conducted and the results are very interesting. Nonetheless, I have a major concern. These data relate exclusively to SNC DA neurons but all rationale including introduction, discussion and all references relate to VTA DA neurons.

In order to publish this set of data as is, the authors should rewrite the introduction and discussion focusing on the effects of chronic stress on SNC dopaminergic neurons. Another possibility would be to keep this introduction / discussion (with minor revision) and combine it with data regarding VTA dopaminergic neurons. I agree that there is a continuum between the VTA and the CNS, but the lack of mention of the differences between the dopaminergic neurons of the VTA and the SNC is very surprising. Furthermore, no mention is made of the literature concerning the differences induced by chronic stress on subpopulations of VTA dopaminergic neurons according to their projection target (NAC vs PFC for example).

Another point concerns the parallels between L-yoked animals and the chronic mild stress model and LOC animals and the social defeat model.The main differences between the chronic mild stress model and the social defeat model are the nature of the stress (environmental vs social) and not the predictability of the stress. In the case of this study, the authors revealed an increased activity of SNC DA neurons in LOC and L-yoke mice and in both groups, the mice underwent environmental stress. This is in contrast to the results obtained on VTA DA neurons from animals that have undergone chronic mild stress or a learned helplessness paradigm and where the electrophysiological parameters of VTA dopaminergic neurons activity is decreased. Perhaps this is because the data in this study relates to SNC neurons instead of VTA neurons?

## Author Response

Dear Dr. Lee

We thank you and the two reviewers for the valuable comments and suggestions

on our manuscript eN-TNWR-0044-21X titled “Stress controllability modulates basal

activity of dopamine neurons in the substantia nigra compacta” which we previously

submitted to eNeuro. We have tried our very best to address all issues mentioned in

the comments. These are included in the attached manuscript. Point-to-point answers

to the comments are listed below.

Responses to Reviewing Editor:

1. Most importantly, the literature into which the study is contextualised and

interpreted relates exclusively to the effects of chronic stress on VTA

dopaminergic neurons. With the current set of data on SNc dopaminergic

neurons (and without recording of VTA dopaminergic neurons), there is a

substantial and important disconnect between the literature and the data.

This needs to be addressed by refocussing the manuscript on the SNc and

acknowledging/discussing the differences between the SNc and VTA. In

particular, the results should be interpreted in relation to established

functions and behaviours linked to the SNc.

Response:

Thank you very much for this constructive suggestion.

The revised manuscript was focused on the changes of activity in SNc DA

neurons in LOC model and the differences between the SNc and VTA were discussed

to interpret the results obtained in the present study. The hypothesis that stress

controllability is relevant to the activity of SNc DA neurons was proposed based on

the functions of SNc DA neurons and previous related findings in VTA. Therefore,

the differences between the SNc and VTA were discussed that may lead to different 2

2

roles in the modulation of behavior. (lines 31-73; 438-456)

2. A second, related, major comment is that the manuscript has an explicit

focus on the comparison between the LOC model and classical stress models.

This comparison is somewhat weakened by the absence of additional

behavioural measures (or at least robust discussion in relation to previous

characterisation of the model). Therefore, the manuscript should be

substantially revised either to reduce such a comparative emphasis, or to add

more robust discussion. This relates explicitly to the above comment about

interpretation of the results in relation primarily to SNc behaviours.

Response:

Thank you for this constructive suggestion.

In revised manuscript, the comparison emphasis between the LOC model and

classical stress models was reduced rather emphasizing responses of SNc DA neurons

to the different stress controllability per se. Of course, the classical behavioral tests

for assessing stress-induced depression-like behaviors including sucrose preference

test and forced swimming test were also used again. The results are consistent with

our previous findings that have been published, that LOC mice losing control over

shock exhibited a significant reduction in appetite to natural reward and a more

serious despair-like behavior, but ES mice acquiring control over shock did not

exhibit similar reduction and behavioral deficit. These reproducible results in

behavioral tests also were added in main text, but the figure was added as an extended

data supporting Figure 1, due to the present study focused on the new results in LOC

model. (lines 287-292)

Accordingly, the main text was revised as follows:

Results:

----"Depression-like behaviors were also assessed by using sucrose preference test 3

3

and swimming forced test. The results were consistent with the previous findings that

LOC mice exhibited a significant reduction in appetite to natural reward and a more

serious despair-like behavior, but ES mice did not exhibit similar reduction and

behavioral deficit (Yao et al., 2019; Figure 1-1).” (lines 287-292)

Extended data:

Figure 1-1. Loss of control over shock produced significant depression-like behaviors. (A) In

sucrose preference test, LOC mice exhibited a significant reduction in sucrose preference index.

Preference index is calculated as the ratio of the number of licks for the sucrose solution to the

total number of licks. (B) In forced swimming test, mice in both the LOC and the L-Yoked groups

exhibited significantly increased immobile time compared with both ES and NS mice, and the

LOC group showed more increase than the L-Yoked group. *p < 0.05, **p < 0.01, ***p < 0.001.

Error bars represent SEM. LOC: loss of control over shocks; L-Yoked: yoked to LOC; ES: escape

shock; NS: no shock. NS: n = 8, L-Yoked: n = 8, LOC: n = 8, ES: n = 6.

Reference:

Yao, L., Li, Y., Qian, Z., Wu, M., Yang, H., Chen, N., . . . Ren, W. (2019). Loss of control over mild aversive

events produces significant helplessness in mice. Behav Brain Res, 376, 112173.4

4

Responses to Reviewer #1:

1. In this study the authors do not show their model results in classical stress

induced behaviors, rendering their proclaimed comparisons with chronic

stress models difficult to believe. Their claim would be made stronger with

additional metrics such as reduced preference for sucrose or social

interaction, enhanced risk aversion metrics like elevated plus maze, novelty

suppressed feeding, or a light-dark box, rather than another learning-based

shuttle-box test.

Response:

We are sorry without reporting the classical behavioral results of LOC model.

In revised manuscript, the classical behavioral tests for assessing stress-induced

depression-like behaviors including sucrose preference test and forced swimming test

were also used again. The results are consistent with our previous findings that have

been published, that LOC mice losing control over shock exhibited a significant

reduction in appetite to natural reward and a more serious despair-like behavior, but

ES mice acquiring control over shock did not exhibit similar reduction and behavioral

deficit. These reproducible results in behavioral tests also were added in main text,

but the figure was added as an extended data supporting Figure 1, due to the present

study focused on the new results in LOC model. In addition, because the changes of

SNc DA neurons in LOC model were concerned in the present study, the comparison

emphasis between the LOC model and classical stress models was reduced. (lines

287-292)

Accordingly, the main text was revised as follows:

Results:

----"Depression-like behaviors were also assessed by using sucrose preference test

and swimming forced test. The results were consistent with the previous findings that 5

5

LOC mice exhibited a significant reduction in appetite to natural reward and a more

serious despair-like behavior, but ES mice did not exhibit similar reduction and

behavioral deficit (Yao et al., 2019; Figure 1-1).” (lines 287-292)

Extended data:

Figure 1-1. Loss of control over shock produced significant depression-like behaviors. (A) In

sucrose preference test, LOC mice exhibited a significant reduction in sucrose preference index.

Preference index is calculated as the ratio of the number of licks for the sucrose solution to the

total number of licks. (B) In forced swimming test, mice in both the LOC and the L-Yoked groups

exhibited significantly increased immobile time compared with both ES and NS mice, and the

LOC group showed more increase than the L-Yoked group. *p < 0.05, **p < 0.01, ***p < 0.001.

Error bars represent SEM. LOC: loss of control over shocks; L-Yoked: yoked to LOC; ES: escape

shock; NS: no shock. NS: n = 8, L-Yoked: n = 8, LOC: n = 8, ES: n = 6.

Reference:

Yao, L., Li, Y., Qian, Z., Wu, M., Yang, H., Chen, N., . . . Ren, W. (2019). Loss of control over mild aversive 6

6

events produces significant helplessness in mice. Behav Brain Res, 376, 112173.

2. The authors do not sufficiently justify or contextualize their results in terms

of SN DA neurons. As many of their sources focus on VTA neurons, what

implication does this have on the field? Are stress-induced behavioral deficits

just failure to coordinate actions?

Response:

Thank you for this valuable comment, which helped us to improve theoretical

grounding and deeply rethink the meaning of data obtained in the present study.

The present study focused on the effects of stress controllability on SNc DA

neurons. Therefore, the manuscript has been done major revision by refocusing the

manuscript on the SNc and discussing the differences between the SNc and VTA.

Numerous previous studies have demonstrated that a variety of stress regulates the

activity of VTA DA neurons, but it also remains controversial. In addition, previous

study has reported that similar roles of the SNc and VTA DA neurons participates in

affective processes. Those findings in VTA are inspiration for the hypothesis

proposed in the present study. Thus, the changes of activity in SNc DA neurons may

be reflected in affective processes regulated by stress controllability rather than its

simple coordinate action, and these changes may contribute to different roles in the

modulation of behavior. (lines 31-73, 439-457)

3. The authors do not discuss or hypothesis the relevance of the bursting

phenomenon vs increased firing rate for stress-induced behaviors.

Response:

Thank you for your reminding.

In revised manuscript, the relationship between the bursting and firing rates for

stress-induced behaviors was discussed in the main text. (lines 471-478)7

7

Accordingly, the main text was revised as follows:

Results:

----"The burst firing pattern, produced on a timescale of hundreds of milliseconds,

plays a key role in encoding salience signals via phasic release of DA, while the

single spike firing pattern depends on the spontaneous firing of DA neurons with

slower changes in tonic levels of DA (Grace et al., 2007). Ih not only regulates the

spontaneous firing of DA neurons, but also regulates the transition of firing mode

between tonic and phasic firing (Cao et al., 2010). Changes in tonic level of DA can

directly influence the magnitude of the phasic DA response regulated by bursting

firing in behavior (Floresco et al., 2003).” (lines 471-478)

In addition, our results also showed that there was a significant positive

correlation between bursting and firing rates of SNc DA neurons. The figure is shown

below:

Reference:

Cao, J. L., Covington, H. E., Friedman, A. K., Wilkinson, M. B., Walsh, J. J., Cooper, D. C., . . . Han, M. H.

(2010). Mesolimbic dopamine neurons in the brain reward circuit mediate susceptibility to

social defeat and antidepressant action. J Neurosci, 30(49), 16453-16458.

Floresco, S. B., West, A. R., Ash, B., Moore, H., & Grace, A. A. (2003). Afferent modulation of dopamine

neuron firing differentially regulates tonic and phasic dopamine transmission. Nat Neurosci,

6(9), 968-973.

Grace, A. A., Floresco, S. B., Goto, Y., & Lodge, D. J. (2007). Regulation of firing of dopaminergic

neurons and control of goal-directed behaviors. Trends Neurosci, 30(5), 220-227.8

8

4. No exclusion criteria are described in the manuscript

Response:

Thank you for your carefully reading and reminding. The exclusion criteria were

added in main text. (lines 240-242)

Accordingly, the main text was revised as follows:

Results:

----"Mice were excluded from statistical analysis when they learned to use various

gestures or actions to avoid shock without nose poking, and the tip of optical fiber is

not in the SNc.” (lines 240-242)

Responses to Reviewer #2:

1. These data relate exclusively to SNC DA neurons but all rationale including

introduction, discussion and all references relate to VTA DA neurons. In order to

publish this set of data as is, the authors should rewrite the introduction and

discussion focusing on the effects of chronic stress on SNC dopaminergic

neurons. Another possibility would be to keep this introduction / discussion

(with minor revision) and combine it with data regarding VTA dopaminergic

neurons. I agree that there is a continuum between the VTA and the CNS, but

the lack of mention of the differences between the dopaminergic neurons of the

VTA and the SNC is very surprising. Furthermore, no mention is made of the

literature concerning the differences induced by chronic stress on

subpopulations of VTA dopaminergic neurons according to their projection

target (NAC vs PFC for example).

Response:

Thanks for your constructive suggestion. 9

9

The revised manuscript was focused on the changes of activity in SNc DA

neurons in LOC model and the differences between the SNc and VTA were discussed

to interpret the results obtained in the present study. Due to previous studies focused

on the effects of chronic stress on SNc DA neurons, hinging on the type, duration,

intensity, and controllability of stressors, the firing activity of DA neurons in SNc

involved in stress controllability is unclear. Therefore, the hypothesis was proposed

that the stress controllability regulated affective processes may have an influence on

the firing activity of SNc DA neurons. Indeed, the SNc and VTA are not completely

separated not only in anatomy, but also in function. Both the SNc and VTA DA

neurons are similarly involved in affective processes linked to stress. Thus, the

differences between the SNc and VTA were discussed to interpret their different roles

in the modulation of behavior. (lines 31-73, 438-456).

2. Another point concerns the parallels between L-yoked animals and the

chronic mild stress model and LOC animals and the social defeat model. The

main differences between the chronic mild stress model and the social defeat

model are the nature of the stress (environmental vs social) and not the

predictability of the stress. In the case of this study, the authors revealed an

increased activity of SNC DA neurons in LOC and L-yoke mice and in both groups,

the mice underwent environmental stress. This is in contrast to the results

obtained on VTA DA neurons from animals that have undergone chronic mild

stress or a learned helplessness paradigm and where the electrophysiological

parameters of VTA dopaminergic neurons activity is decreased. Perhaps this is

because the data in this study relates to SNC neurons instead of VTA neurons?

Response:

Thanks for your reminding.

Indeed, previous studies have demonstrated that the chronic mild stress and the

social stress play different roles in regulating activity of VTA DA neurons, that may 10

10

contribute to different behaviors. In the present study, mice of LOC group losing

control over footshock and those of L-Yoked group receiving the identical footshock

to lack of control exhibited significantly increased activity of SNc DA neurons. The

difference between the SNc and VTA DA neurons in response to stress that may

result in behavioral deficit, imply functional diversion of midbrain DA neurons, and

therefore different roles in the modulation of behavior. Therefore, in the future, it is

important to pay attention to distinguish DA subpopulations involved in behavioral

deficit induced by distinct stress.

Thank you very much again!

Sincerely yours,

Wei Ren, Ph.D.

Professor in Psychology and Neuroscience

MOE Key Laboratory of Modern Teaching Technology

Shaanxi Normal University
