# Supplementary figures and images for: Stress Controllability Modulates Basal Activity of Dopamine Neurons in the Substantia Nigra Compacta
Source: eNeuro. 2021 Jun 15;8(3):ENEURO.0044-21.2021. doi: 10.1523/ENEURO.0044-21.2021 (PMC8211467; doi:10.1523/ENEURO.0044-21.2021)

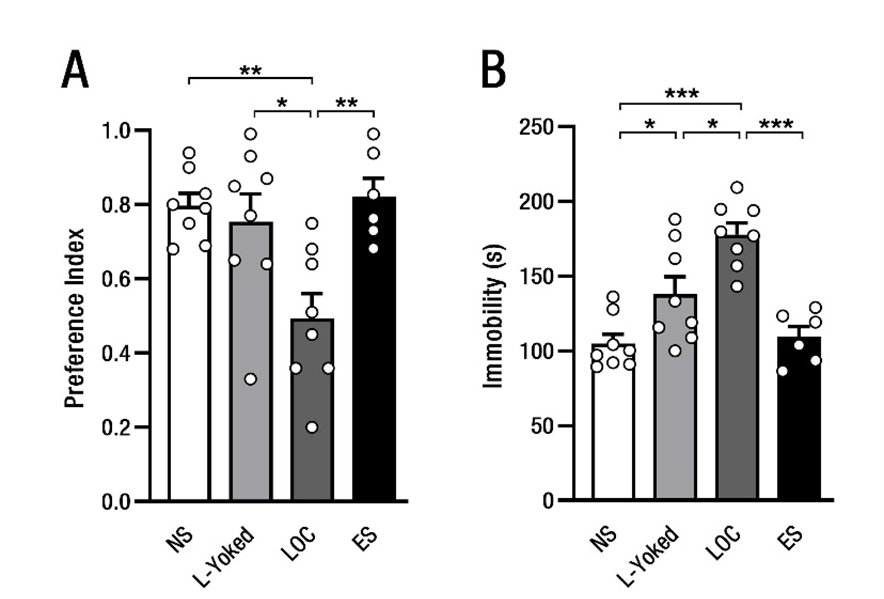

Supplement: Extended Data Figure 1-1 — LOC over shock produced significant depression-like behaviors. A, In the SPT, the LOC group exhibited a significant reduction in sucrose preference index, as compared with the other three groups. Preference index is calculated as the ratio of the number of licks for the sucrose solution to the total number of licks. B, In the FST, both the LOC and the L-Yoked groups exhibited significantly longer immobile time than the ES and the NS groups, respectively. In addition, the LOC group showed even longer immobile time than the L-Yoked group; *p < 0.05, **p < 0.01, ***p < 0.001. Error bars represent SEM. LOC: loss of control over shock; L-Yoked: yoked to LOC; ES: escape shock; NS: no shock. NS: n = 8, L-Yoked: n = 8, LOC: n = 8, ES: n = 6. Download Figure 1-1, TIF file. [file enu-eN-NWR-0044-21-s01.tif]
